# Self–other discrimination in face recognition depending on personal familiarity: investigating a sample consisting of Japanese and Han Chinese women

**DOI:** 10.1007/s00426-026-02335-0

**Published:** 2026-07-06

**Authors:** Wenxiao Fang, Kenta Eguchi, Takao Fukui

**Affiliations:** 1 Department of Computer Science, Graduate School of Systems Design, Tokyo Metropolitan University, Hino, Tokyo Japan; 2 Faculty of Systems Design, Tokyo Metropolitan University, 6-6 Asahigaoka, Hino, Tokyo, 191-0065 Japan

## Abstract

**Abstract:**

This study explored how the inclusion of others in the self during face recognition is modulated by personal familiarity, cultural background within East Asia, and autistic traits. Female Japanese and Han Chinese university students completed the Autism-Spectrum Quotient (AQ) questionnaire and performed a self–other discrimination task using images of their own face morphed with that of a friend or stranger of the same sex. Thresholds and widths were calculated by fitting a psychometric function to each participant's responses. In both groups, the threshold in the self–friend morphing condition was significantly lower than in the self–unknown morphing condition, and the width in the self–friend morphing condition was significantly larger than in the self–unknown morphing condition, supporting the concept of inclusion of close others in the self and indicating a blurred boundary between the self and close friends. Regarding cultural differences, Japanese participants showed a higher threshold than Chinese participants in the self–unknown condition, whereas no group difference was observed in the self–friend condition. Furthermore, multiple regression analyses revealed that social skill, a subscale of the AQ, was significantly associated with the thresholds in the self–unknown condition, but not in the self–friend condition. This study extends prior research by demonstrating that personal familiarity strongly modulates self–other discrimination in East Asian females and highlights the importance of considering individuals’ autistic traits and cultural differences, even within the same cultural region, in face recognition research.

**Statement of public significance:**

Recognizing faces is vital for social life. In Japanese and Han Chinese women, close friendships blur the self–other boundary, and the recognition of unknown faces is linked to social skills. When judging blended images made from their own face and a stranger’s face, Japanese participants need more of their own facial features than Chinese participants do when deciding that the face is their own. These findings deepen our understanding of social cognition in diverse societies.

**Supplementary Information:**

The online version contains supplementary material available at https://doi.org/10.1007/s00426-026-02335-0.

## Introduction

The human face is an essential social source, and face recognition plays a major role in self–other discrimination (e.g., Keenan et al., 2000), which is pivotal in social interactions (e.g., Decety & Sommerville, 2003; Jeannerod, 2004; Ruby & Decety, 2001).

### Effects of personal familiarity on face recognition

The individuals with whom we engage in face-to-face social interactions in daily life can be categorized from strangers to spouses, according to the degree of their “mental closeness” to oneself (i.e., personal familiarity). In addition to differences in face recognition between the self and others, the processes involved in recognizing personally familiar faces have been studied at both the behavioral and neural levels, along with visually familiar, famous, and experimentally learned faces within a framework of the concept of familiarity (e.g., Natu & O’Toole, 2011; Ramon & Gobbini, 2018; Sugiura, 2014, for reviews).

At the behavioral level, Bortolon et al. (2018) used a delayed-matching task with ambient images to show that participants recognized their own faces faster and more accurately than famous or unknown faces, but not more quickly than a friend’s face. Friends’ faces were also recognized better than unknown faces, with no significant difference between friends and famous faces. These findings support the idea of a familiarity continuum in face recognition, ranging from unknown to highly familiar faces, including one’s own. Furthermore, Visconti di Oleggio Castello and Gobbini (2015) showed that participants reliably made correct and rapid saccades (around 180 ms from stimulus onset) to their friends’ faces when the distractor (i.e., another potential target) was an unfamiliar face. They proposed a specific and rapid detection process for familiar faces. At the neural level, Visconti di Oleggio Castello et al. (2017) conducted multivariate pattern analyses on fMRI data to dissociate familiarity and identity information and revealed that identity-independent information about familiarity could be decoded in extended system areas such as the temporo-parietal junction, precuneus, and the medial prefrontal cortex, for inferring socially relevant information from faces, such as the retrieval of person knowledge, and in the dorsal and anterior core system areas such as the middle temporal gyrus/superior temporal sulcus, anterior fusiform cortex, and the inferior frontal gyrus, for analyzing the visual appearance of faces (see also Kovács, 2020, for a review). Furthermore, Campbell et al. (2020) found that identity-specific responses in the bilateral occipito-temporal cortex, measured using fast periodic visual stimulation with EEG (FPVS-EEG), were strongest for one’s own face, intermediate for a friend’s face, and weakest for a stranger’s face, demonstrating a graded effect of personal familiarity. They also revealed that familiar faces (one’s own and friends) elicited distinct activity over the posterior midline cortex, suggesting the engagement of post-perceptual person knowledge processes.

In addition to the above, other studies have employed morphing techniques (e.g., Sutherland et al., 2017, for a review), whereby the blending ratio between two faces is altered to synthesize a single face to investigate self–other discrimination in face recognition (Keenan et al., 1999; Ketay et al., 2019; Kircher et al., 2001; Ma & Han, 2012; Tsakiris, 2008; Uddin et al., 2008). For example, Ketay (2019) revealed longer reaction times for self–friend morphs than for self–celebrity or friend–celebrity morphs in a self-identification task. They explained this modulation of personal familiarity in face recognition as the “inclusion of others in the self.” This social psychological concept refers to psychological closeness between the self and close others, in which the self overlaps with close others in terms of resources, perspectives, and characteristics (Aron et al., 1991). Specifically, a romantic partner is regarded as a part of the self, and people do, indeed, experience “self–partner confusion” concerning personality traits (Aron et al., 1991; Mashek et al., 2003). Thus, Ketay et al. (2019) demonstrated that this inclusion of others in the self could also occur in self–other discrimination for face recognition, implying this inclusion could expand to the visual perception domain.

### Autistic traits as individual differences in self–other face discrimination

When considering self–other discrimination for face recognition in social interactions, it is essential to account not only for the relationship between the self and others captured by personal familiarity, but also for the individual traits involved in face recognition. It has been reported that individuals with autism spectrum disorder (ASD) exhibit atypical face processing (e.g., Dawson et al., 2005; Tanaka & Sung, 2016; Tang et al., 2015; Weigelt et al., 2012; Zhang et al., 2025, for reviews). In addition to group-comparison studies of individuals with ASD and their typically developing peers, research has examined whether autistic traits in non-clinical populations, as measured by the Autism-Spectrum Quotient (AQ; Baron-Cohen et al., 2001), are related to individual differences in face-recognition ability. For example, Halliday et al. (2014) found, using an immediate face-memory task that required participants to match a briefly presented target face to one of three probe faces on the basis of identity, that higher AQ scores were significantly associated with poorer face-recognition performance. Furthermore, Davis et al. (2017) showed that autistic-trait subclusters were differentially associated with face processing. Higher AQ-Social (socially relevant traits) scores were associated with poorer face-recognition performance in women from the general population, significantly so on the Cambridge Face Memory Task (Duchaine & Nakayama, 2006), whereas higher AQ-Attention (attention-to-detail traits) scores were associated, across the full sample of men and women, with an increased number of fixations to the eyes and longer dwell time on the eyes during face learning. In separate mediation models, higher AQ-Attention was indirectly related to better face-recognition performance via each of two eye-looking measures. The relationship between autistic traits and self–other face discrimination is also relevant to broader discussions of self-referential processing in ASD, where atypical self-related cognition and atypical coordination between self- and other-related representations have been suggested (Baron-Cohen, 2005; Frith & de Vignemont, 2005; Frith & Happé, 1999; Lombardo et al., 2010; Lombardo & Baron-Cohen, 2010).

In this context, Chakraborty and Chakrabarti (2015) engaged participants in a self–other discrimination task using morphed face images of participants and unfamiliar persons within a sample of the general population. They found no significant association between AQ scores and task performance. However, they did not apply a task using morphed images of participants and personally familiar persons, so the extent to which autistic traits modulate self–other discrimination, especially in the context of high personal familiarity, remains unclear. In addition, their analysis did not examine psychometric threshold estimates in the manner adopted in the present study, leaving open the possibility that autistic traits may be related specifically to the point at which the self–other categorization becomes ambiguous. The present study addresses this gap by examining whether AQ subscale scores are associated with psychometric indices of self–other face discrimination in both self–friend and self–unknown morphing conditions.

### Cultural variation in face processing within East Asia

Cultural differences are also known to shape face processing. For example, an eye tracking study by Blais et al. (2008) revealed that East Asians fixate more on the center of faces and less on the eyes and mouth areas than Western Caucasians do (see also Blais et al., 2021; Caldara, 2017, for reviews). Such cross-cultural research has traditionally focused on comparisons between East Asian and Western populations (e.g., Markus & Kitayama, 1991; Matsumoto & Ekman, 1989; Nisbett & Miyamoto, 2005), and less attention has been paid to comparisons between different countries within the same cultural group (e.g., Japan and China in East Asia), with a few exceptions (Cao et al., 2017; Mao & Daibo, 2008; Ozono, 2019). To our knowledge, no previous research has examined cultural modulation in self–other discrimination of face recognition considering personal familiarity.

In comparisons between Western and East Asian cultures, cultural psychology has often characterized Western societies as relatively individualistic and independent, whereas East Asian societies have been described as more collectivistic and interdependent (e.g., Markus & Kitayama, 1991). Within this framework, Japanese and Chinese participants are frequently treated as members of a single, relatively homogeneous East Asian or collectivistic culture. However, such a characterization may obscure important differences within East Asia. Dien (1999), for example, questioned whether Japanese and Chinese sociality should be subsumed under a single collectivistic label and argued that the two groups differ in their characteristic social orientations, describing Japanese sociality as more peer-group oriented and Chinese sociality as more authority-directed. From this perspective, Japanese and Han Chinese participants should not be treated simply as members of a single, undifferentiated East Asian culture.

Ozono (2019) reported that general trust, defined as trust directed toward people in general, is higher in China than in Japan. In the self–unknown condition, participants judge morphed faces involving the self and another person with whom no personal relationship has been established. This condition is, therefore, particularly relevant to general trust-related orientations toward others. Based on differences in general trust, we reasoned that individuals from cultures with higher general trust would adopt a more inclusive criterion for distinguishing self from other. In the context of a face morphing task, this would suggest that individuals with higher general trust may be more inclined to accept ambiguous blends of the self and an unfamiliar other as the self. Thus, we hypothesized that culturally shaped processes of self–other face discrimination would be differentially modulated by personal familiarity across Chinese and Japanese participants. Specifically, in the self–unknown condition, Chinese participants would require a lower proportion of the self in a morphed face to identify it as the self than Japanese participants would, whereas no clear group difference would emerge in the self–friend condition.

### Research questions of the present study

The studies reviewed above suggest that self–other discrimination in face recognition is influenced by multiple factors, including the degree of personal familiarity with others, individual differences related to autistic traits, and culturally shaped orientations toward the self and others. The present study integrates these perspectives by examining self–other face discrimination in Japanese and Han Chinese female participants using morphed images of participants’ own faces with either a close friend or an unfamiliar person of the same sex, while assessing individual differences in autistic traits.

Specifically, we explore the following hypotheses in the present study:

1. Self–other face discrimination differs as a function of personal familiarity;
2. Autistic traits are associated with individual differences in psychometric indices of self–other discrimination; and
3. Self–other discrimination may be differentially modulated by cultural background within East Asia.

## Materials and methods

### Participants

The sample size was determined by conducting a power analysis using G*Power 3 (Faul et al., 2007). Assuming a medium effect size of f = 0.25, α = 0.05 and power (= 1 - β) = 0.80 were set to test within–between interactions using a repeated measures ANOVA (i.e., two ethnic groups [Japanese, Chinese] as a between-participants factor and image set [self–friend, self–unknown] as a within-participant factor). The power analysis indicated that a total sample size of 34 (17 for each group) was needed. We recruited 37 female participants for the face recognition task. Because the first author (WF) contributed her facial photograph for stimulus preparation but did not participate in the task, the final set of photographed individuals comprised 38 women (18 Japanese and 20 Han Chinese), forming 9 Japanese friendship pairs and 10 Chinese friendship pairs, with WF included in one of the Han Chinese pairs (see below). Participants brought a female friend of a similar age for the photography session. Participants were self-reported right-handed undergraduate or graduate students recruited from Tokyo Metropolitan University. All received course credit or payment for their participation.

Of these participants, two Chinese participants were excluded because of their outlier performances, leaving 35 participants (18 Japanese [mean age ± SD: 20.8 ± 0.9, range = 19–22] and 17 Han Chinese [mean age ± SD: 23.4 ± 2.5, range = 19–29]) for inclusion in the analysis. Although we noted a significant age difference between the Japanese and Chinese groups [*t*(20.152) = -4.036, *p* < 0.001] (Figure S1), the influence of such a difference among women in their early 20s on the ability to recognize faces was assumed to be marginal. Rather, according to the purpose of this study, which was comparing self–friend morphing images with self–unknown morphing images, we controlled for friendship relationship length, calculated as the total duration of the friendship in months divided by 12, between the two groups (Japanese [mean years ± SD: 2.6 ± 0.7, range = 1.67–3.75], Chinese [mean years ± SD: 2.7 ± 0.8, range = 1.58–3.75]), and no significant difference in this length was found between the two groups [*t*(33) = -0.125, *p* = 0.901].

No participants had facial markings such as obvious moles or birthmarks, which would make them easily identifiable. All had normal or corrected-to-normal vision, and none had any motor or sensory abnormalities. This study was approved by the ethics committee of Tokyo Metropolitan University’s Hino Campus (2018-No.267), and all participants provided their written informed consent according to the Declaration of Helsinki. All participants were naive to the purpose of the experiment. The experiment was performed before the onset of the COVID-19 pandemic (i.e., December 2018–December 2019).

## Stimuli and procedure

### Photographing and editing face images

We photographed all participants’ faces at least one week prior to the face recognition task. Each pair came to together to have their photos taken, a task that took roughly 10 min per person. Photographs were taken in a room under standard lighting conditions, with care taken to minimize the influence of outside daylight, using a digital camera (PowerShot SX620 HS, Canon, Japan). The lighting conditions were not photometrically calibrated. Participants were asked to show a neutral expression for the photograph. Full-color photographs were used, and no additional luminance normalization was applied. The images were then edited to remove non-facial features, so that only an oval image of their facial features remained. Their hair and ears were cropped from the photos. To create the unknown female image for each group, one Japanese and one Han Chinese person were selected. Neither was acquainted with any of the participants, and both were close in age to the participants (aged 23 and 24, respectively).

A set of face stimuli was created for each participant, by morphing each participant’s face with a friend’s face or an unknown one, using Abrosoft FantaMorph software. The morphed face stimuli in each self–friend and self–unknown condition included 15 morphed levels (i.e., 0, 5, 10, 20, 30, 40, 45, 50, 55, 60, 70, 80, 90, 95 and 100% of the self). The response data obtained at these 15 morph levels were then fitted with psychometric functions using Psignifit 4 (see below). Previous face recognition studies using facial morphs have often used morph series with uniform increments, for example, 11 images in 10% increments or 21 images in 5% increments (e.g., Keyes, 2012; Kircher et al., 2001). However, Psignifit 4 does not require equally spaced morph levels. Rather, fitting depends on whether the selected stimulus levels adequately sample the region of the continuum over which responses change, and the default priors for threshold and width are set with reference to the overall range of the stimulus levels and the intervals between them (Schütt et al., 2016). We therefore sampled the middle of the continuum more densely (40–60% self) to better capture the expected transition region, and included 5% and 95% self to supplement the endpoints with observations close to the lower and upper asymptotic portions of the psychometric curve. This arrangement also allowed more fine-grained sampling in regions of the self–other continuum that were either clearly identifiable (0–10% self and 90–100% self) or relatively ambiguous (40–60% self).

To correspond with participants’ daily experiences of looking at their own faces and their friends’ faces, mirror-reversed photographs of the participants themselves (e.g., Kircher et al., 2000, 2001) and non-mirror-reversed photographs of others (i.e., friends and unknowns) were used. This manipulation of participants’ own images is consistent with findings by Suchow et al. (2024) showing that mirror‑reversed self‑images, compared with non‑reversed self-images, are perceived as more similar to individuals’ internal representations of their own faces.

### Face recognition task

A laptop PC (ZenBook Pro UX550VD-7700. AsusTek Computer Inc., Taipei, Taiwan; 15.6 inch, screen resolution = 3840 × 2160 pixels) was used to present the stimuli and for data acquisition. Participants looked at the laptop screen from a distance of approximately 60 cm while seated comfortably on a chair in front of a table on which the laptop was placed. A chinrest was not used, and short breaks were provided, as needed, to reduce fatigue and maintain a stable posture. At the beginning of the trial, a white cross (i.e., a fixation point, 1.2˚ × 1.2˚) was presented in the center of the display. When participants were ready to begin the trial, they pressed the “space” key on the keyboard. Immediately after pressing the key, a full-color face stimulus (5.7˚ × 7.3˚) was presented in the center of the display. Participants were instructed to decide whether the presented face was their own face or another person’s (i.e., their friend’s face, in the self–friend morphing condition, or an unknown’s face, in self–unknown morphing condition) and to respond as quickly and accurately as possible. Each face stimulus remained on the screen until the participant made a response. After they responded, the fixation cross re-appeared and the next trial began. Face stimuli (with 15 morphing levels) were presented randomly. Participants were instructed to press the “F” key using the index finger of their left hand to respond to their own face’s image and the “J” key using the index finger of their right hand to respond to the image of another person’s face. Keenan et al. (1999) reported that stimuli of one’s own face were identified more rapidly than stimuli of another person’s face, when the participants responded with their left hand. Accordingly, the response indicating the participant’s own face was assigned to the left hand.

This is the first face-recognition experiment launched by our research group. In this initial study, all participants completed the self–friend condition before the self–unknown condition. This fixed order was adopted on the basis of preliminary observations and was intended to minimize possible participant discomfort associated with starting the task with the self–unknown condition. The experiment consisted of 225 trials (15 morphing levels × 15 trials) in each self–friend and self–unknown morphing condition (i.e., a total of 450 trials).

### Questionnaires

In addition to noting how long participant pairs had been friends, we used the Friendship Function Scale (Tanno, 2008) as a supplementary measure to check for possible group differences in friendship strength. This scale aims to assess multiple dimensions of the extent to which a respondent relates to a specific friend. It consists of nine subscales, each of which has five items, resulting in a 45-item questionnaire (e.g., “I think A will be a friend throughout my life”). Participants rated every statement using a 5-point Likert-type scale from 1 (not at all) to 5 (extremely). The maximum possible score is 45, with higher scores indicating a stronger relationship with the friend. Because a validated Chinese version of this scale was not available at the time of data collection, the original Japanese version was used for the Chinese participants. All Chinese participants had considerable ability to understand Japanese (as indicated by the Japanese-Language Proficiency Test) and they were permitted to consult a digital dictionary for unfamiliar words. The mean scores of the Japanese and Chinese groups were 35.62 (SD = 4.60) and 35.51 (SD = 4.80), respectively, and no significant difference was found [*t*(33) = 0.073, *p* = 0.942].

In addition to the instruments detailed above, we used the Japanese (Wakabayashi et al., 2004) and Chinese versions (Wu, 2015) of the Autism-Spectrum Quotient (AQ) (Baron-Cohen et al., 2001) to investigate whether and how autistic traits influence the face-recognition task used in the present study. The AQ consists of five subscales (social skill, attention switching, attention to detail, communication, and imagination). Each subscale has 10 items, resulting in a 50-item questionnaire with a maximum possible score of 50. A score of 33 (the cutoff score in the Japanese version) or higher indicates that the participant could possibly have a clinically significant level of autistic traits. The mean AQ scores of the Japanese and Chinese groups were 19.44 (SD = 4.87) and 19.47 (SD = 6.25), respectively, and no significant difference was noted [*t*(33) = -0.014, *p* = 0.989].

### Data processing and analysis

We calculated the proportion of responses indicating that the presented morphing image was the participant’s own face, and the data for each participant and each condition were fitted with a cumulative normal distribution function with equal asymptotes using Psignifit 4 (Schütt et al., 2016). We determined the threshold and width for each participant and each condition based on the fitted function. The threshold is the level at which the unscaled sigmoid function has a value of 0.5, and the width is the difference between the levels at which the function reaches 0.05 and 0.95 (Schütt et al., 2016). The width value is interpreted as an extent of overlap between the participant’s own face and the representation of another face. A smaller width indicates a reduced overlap between self- and other-representations; that is, a more distinct self-representation (cf. Chakraborty & Chakrabarti, 2015, 2018). Mean values of the threshold and width were entered into a two-way ANOVA with the group (Japanese, Chinese) as a between-participants factor and the morphed face stimuli (self–friend, self–unknown) as a within-participants factor. Bonferroni-corrected post-hoc comparisons were performed when necessary. As a supplementary analysis, threshold and width were also estimated from the same response data using the 11 evenly spaced levels (0–100% self in 10% increments) from the original 15-level set. We then conducted ANOVAs on the 11-level estimates and calculated correlations between the 11-level and 15-level estimates (see Supplementary Information). As the Japanese and Han Chinese groups showed a significant difference in age, we additionally conducted a one-way ANCOVA including age as a covariate to confirm that the group main effect observed in the two-way ANOVA was not attributable to differences in age between the samples.

By pooling the data for the Japanese and Chinese groups, a multiple linear regression and associated stepwise variable selection method were applied to analyze whether and which subscale scores (i.e., social skill, attention switching, attention to detail, communication, imagination) were associated with the threshold and the width. When performing the multiple linear regression, two levels (i.e., self–friend and self–unknown conditions) were set in the experimental design, so α = 0.05/2 = 0.025 was considered to be statistically significant, using the Bonferroni correction.

## Results

**Fig. 1:**
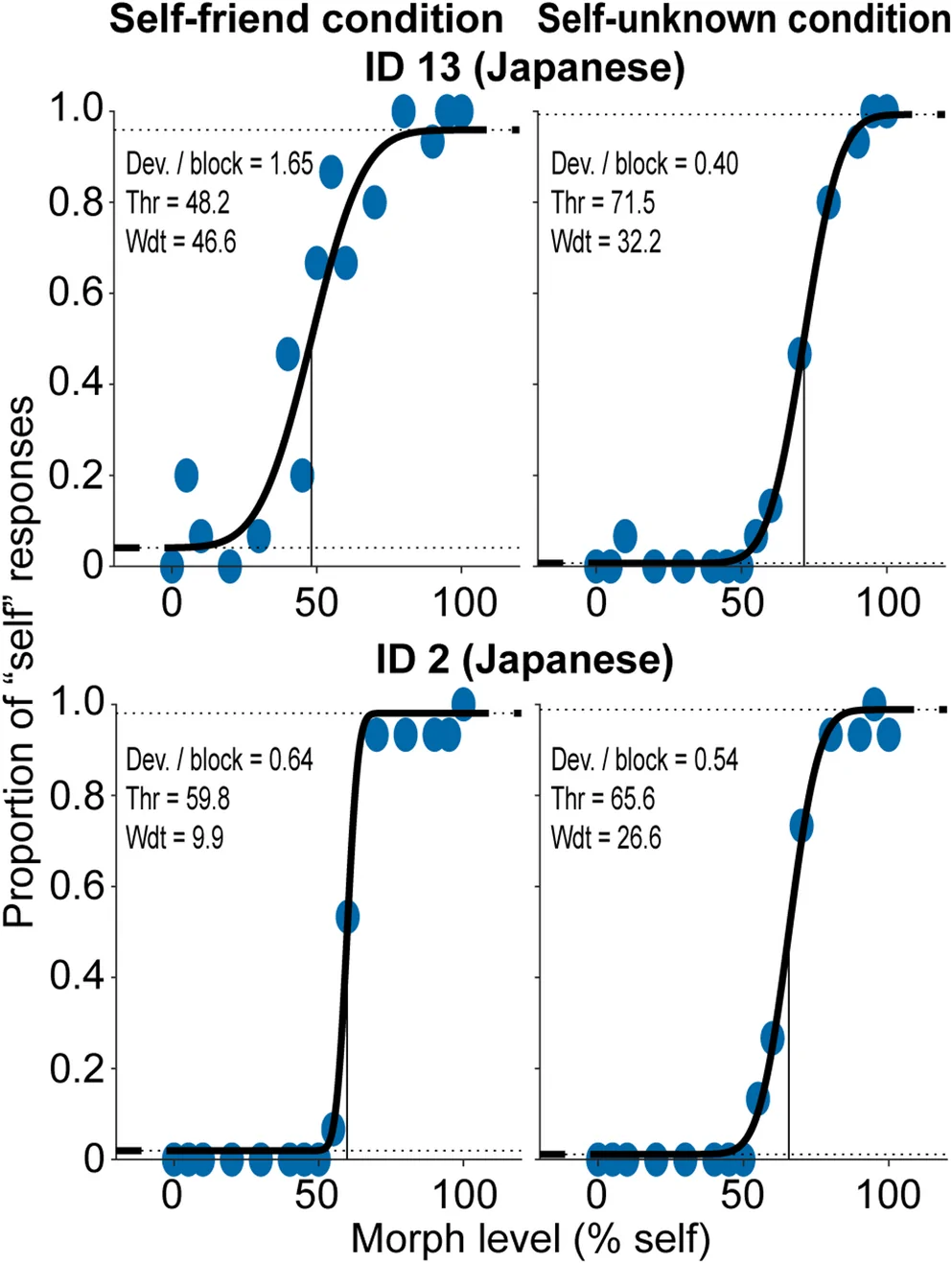
Individual psychometric functions fitted to response data in the self–friend (left) and self–unknown (right) conditions. The top panels show data from the participant with the largest deviance per block, whereas the bottom panels show data from a participant whose deviance per block was close to the median across participants in both conditions. Dev./block, Thr, and Wdt indicate deviance per block, threshold, and width, respectively

In Psignifit 4, deviance per block served as a goodness-of-fit measure, whereas the overdispersion parameter (*η*) indexed residual variability beyond the binomial assumption. According to Schütt et al. (2016), deviance per block values are generally close to 1, with values below approximately 2 considered indicative of well-behaved psychometric data, and *η* equal to zero indicates a binomial observer. For all participants, deviance per block values did not exceed 1.65, and the *η*s were below 0.0001 (see Table S1). Figure 1 presents illustrative individual psychometric functions fitted to the response data. The top row displays fitted psychometric functions for each condition (left: self–friend; right: self–unknown) from the participant who exhibited the largest deviance per block value (1.65) in the self–friend condition. By contrast, the bottom row shows fitted psychometric functions from a participant with deviance per block values (0.64 for the self–friend condition and 0.54 for the self–unknown condition) that were close to the median across participants in both conditions (Mdn = 0.68 for the self–friend condition and Mdn = 0.54 for the self–unknown condition).

### Threshold (Fig. 2A)

We found a significant main effect of the morphed face stimuli [*F*(1, 33) = 43.249, *p* < 0.001, partial *η*^2^ = 0.567], with a lower threshold for the self–friend condition (*M* = 54.6, *SD* = 10.3) than for the self–unknown condition (*M* = 63.3, *SD* = 8.1), while no significant main effect of the group was noted [*F*(1, 33) = 1.545, *p* = 0.223, partial *η*^2^ = 0.045] between the Japanese (*M* = 60.6, *SD* = 9.5) and Chinese (*M* = 57.2, *SD* = 6.6) groups. An additional one-way ANCOVA including age as a covariate indicated that this non-significant main effect of the group remained unchanged after controlling for age [*F*(1, 32) = 2.173, *p* = 0.150]. The interaction between the group and the morphed face stimuli was significant [*F*(1, 33) = 5.868, *p* = 0.021, partial *η*^2^ = 0.151]. Post-hoc comparisons with Bonferroni correction (α = 0.025) revealed a significantly larger threshold in the Japanese group (*M* = 66.5, *SD* = 8.7) than in the Chinese group (*M* = 59.9, *SD* = 5.9) for the self–unknown condition (*p* = 0.014), whereas no significant group difference was observed in the self–friend condition between the Japanese (*M* = 54.7, *SD* = 11.7) and Chinese (*M* = 54.4, *SD* = 9.0) groups (*p* = 0.934). The same overall pattern of results was observed in a supplementary analysis based on an evenly spaced 11-level subset (0–100% self in 10% increments; see Supplementary Information).

### Width (Fig. 2B)

We found a significant main effect of the morphed face stimuli [*F*(1, 33) = 25.038, *p* < 0.001, partial *η*^2^ = 0.431], with a larger width for the self–friend condition (*M* = 38.2, *SD* = 12.7) than for the self–unknown condition (*M* = 28.7, *SD* = 10.9), but a main effect of the group was not significant between the Japanese (*M* = 32.8, *SD* = 9.8) and Chinese (*M* = 34.3, *SD* = 11.4) groups [*F*(1, 33) = 0.175, *p* = 0.679, partial *η*^2^ = 0.005]. An additional one-way ANCOVA including age as a covariate indicated that this non-significant main effect of group remained unchanged after controlling for age [*F*(1, 32) = 1.380, *p* = 0.249]. The interaction between the group and the morphed face stimuli was not significant [*F*(1, 33) = 0.886, *p* = 0.353, partial *η*^2^ = 0.026]. Mean width values were 38.3 (*SD* = 13.6) in the Japanese self–friend condition, 27.2 (*SD* = 9.2) in the Japanese self–unknown condition, 38.1 (*SD* = 12.2) in the Chinese self–friend condition, and 30.4 (*SD* = 12.5) in the Chinese self–unknown condition. The same overall pattern of results was observed in a supplementary analysis based on an evenly spaced 11-level subset (0–100% self in 10% increments; see Supplementary Information).

### Relationship between each performance value (threshold, width) and the AQ subcategory (Fig. 3)

The multiple regression analysis revealed that only the subcategory score for social skill was significantly and negatively correlated with the threshold in the self–unknown condition (*β* = -0.411, *R*^2^ = 0.169, *p* = 0.014), such that higher social skill scores were associated with lower thresholds. No significant associations were found for the remaining AQ subcategory scores (*p*s > 0.426). In addition, none of the other measures, including the threshold in the self–friend condition and the widths in the self–friend and self–unknown conditions, showed significant associations with any AQ subcategory scores. Although Baron-Cohen et al. (2001) do not provide a detailed definition of “social skill,” the content of the items in this subscale suggests that it reflects confidence and ease in social situations, including preferences for social activities and comfort with social interactions (Baker et al., 2024). Accordingly, the social skill subscale can be interpreted as indexing behavioral flexibility in social situations. Therefore, in the self–unknown condition, higher threshold values are linked with greater behavioral flexibility in social situations.

**Fig. 2:**
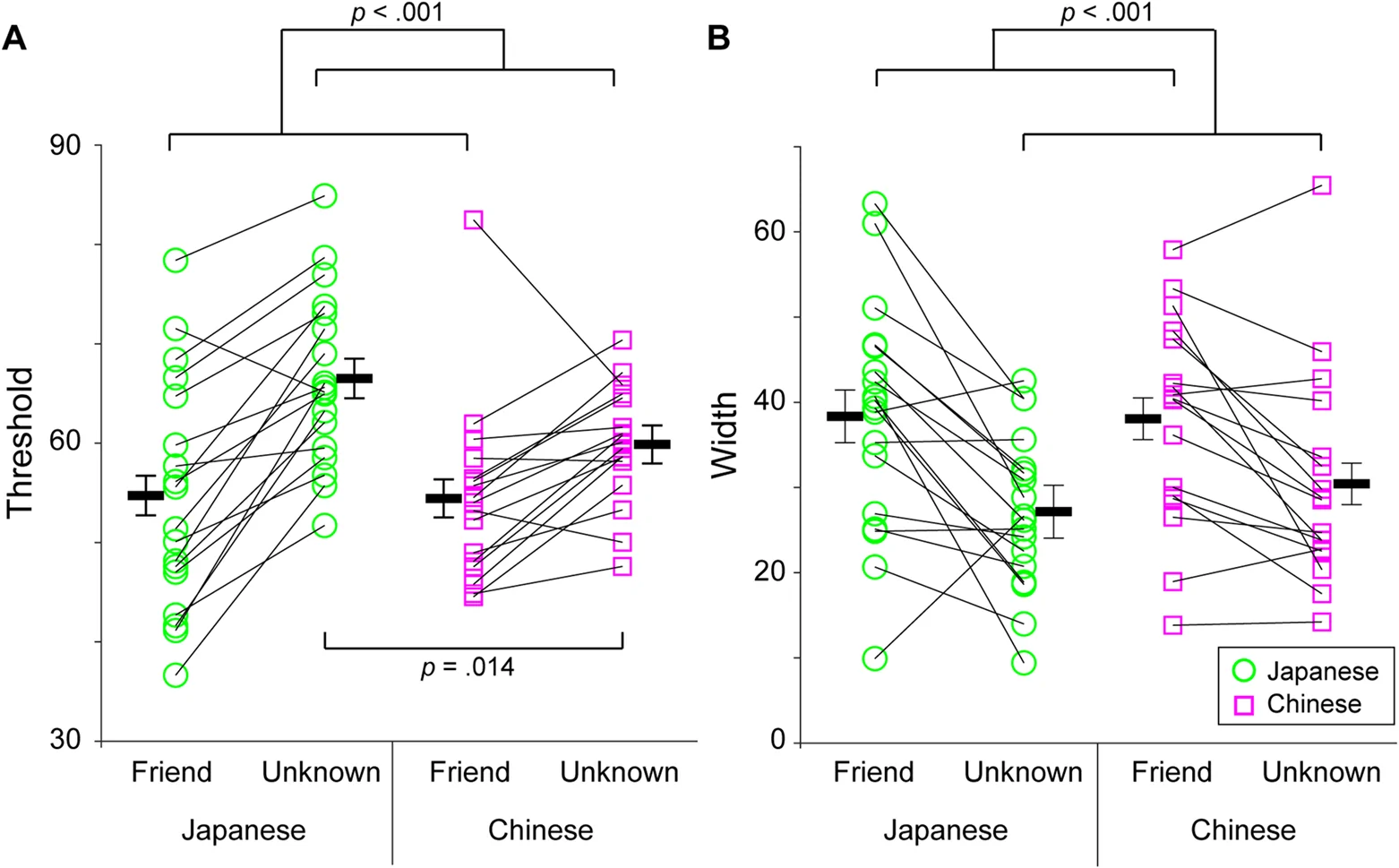
Threshold (**A**) and width (**B**) of self–friend and self–unknown morphing conditions in Japanese and Chinese groups. A significantly lower threshold and larger width in the self–friend morphing condition, compared to the self–unknown morphing condition, was found in both the Japanese and Chinese groups. For threshold, Japanese participants showed a higher value than Chinese participants in the self–unknown condition, whereas no group difference was observed in the self–friend condition. Black bars indicate mean values, and error bars indicate Baguley’s (2012) difference-adjusted normalized 95% confidence intervals

**Fig. 3:**
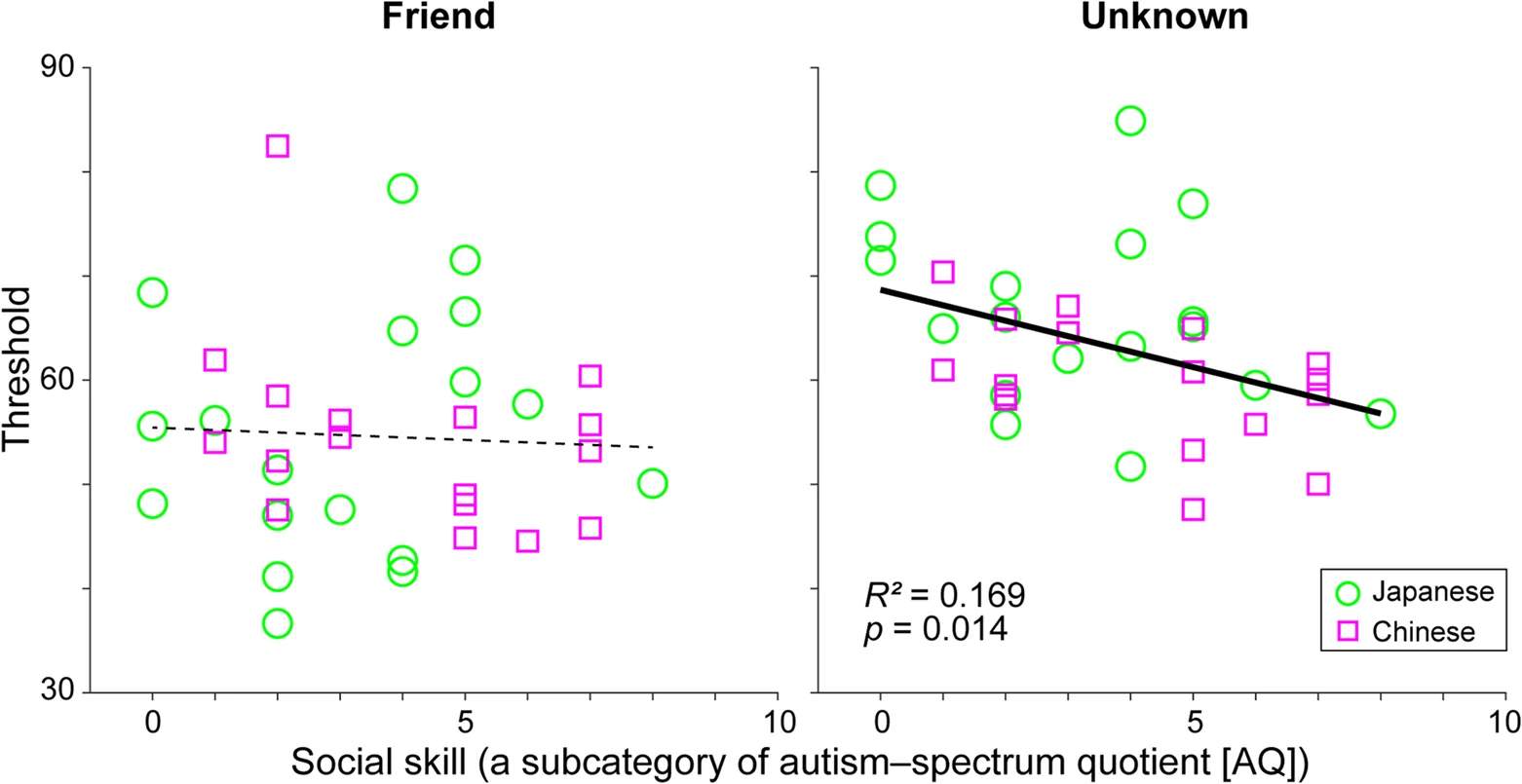
Results of multiple regression analyses of thresholds in the self–friend and self–unknown morphing conditions (based on pooling the data for the Japanese and Chinese groups). Only the social skill subcategory score was significantly correlated with the threshold in the self–unknown condition

## Discussion

This face-recognition study explored whether and how two factors—the extent of same-sex relationship closeness and the degree of autistic traits—modulate self–other discrimination using morphed images in an East Asian population consisting of Japanese and Han Chinese female university students. The results of our psychometric investigation revealed, in both the Japanese and Chinese groups, that (i) the threshold in the self–friend morphing condition (54.6) was significantly lower than in the self–unknown morphing one (63.3), and that (ii) the width in the self–friend morphing condition (38.2) was significantly larger than in the self–unknown morphing condition (28.7) (see Fig. 2). These results indicate that (i) a higher percentage of the self in a morphed photograph is needed to obtain the chance-level (50%) response in the self–unknown morphing condition than in the self–friend morphing condition and that (ii) there was a reduced overlap between the self- and other-representation (i.e., a more distinct self-representation) in the self–unknown morphing condition, in contrast to a vague distinction between the self and a (close) other in the self–friend morphing condition.

One possible account for this pattern is that greater visual exposure to a friend’s face than to an unknown face may result in a more stable and individuated representation of that identity. This exposure-based account could explain the lower threshold in the self–friend condition. However, an account based on differences in visual exposure does not readily explain the increased width in the self–friend condition. If visual exposure primarily strengthened the individuation of the self and the friend as separate identities, this would be expected to facilitate sharper categorical discrimination (i.e., a smaller width), rather than a broader transition range (i.e., a larger width). However, such an expectation is inconsistent with the present pattern. By contrast, the decreased threshold and increased width in the self–friend morphing condition (in comparison with the self–unknown morphing condition) may reflect the inclusion of a close other in the self and a blurring of the boundary between the self and that close friend. This is in line with a previous study analyzing reaction times (Ketay et al., 2019). From this perspective, self-recognition may rely not only on uniquely self-specific facial information but also on facial information incorporated into the self from a close other, while the distinction between the self and a close other becomes less sharply delineated. Recent research (Quintard et al., 2020) has demonstrated that, when using the joint Simon task (Sebanz et al., 2003), the phenomenon of blurring the boundaries between oneself and intimate others also extends to the bodily level (see also Fukui et al., 2020, who used a visual hand discrimination task).

Although six Asian Americans were included in the sample (which consisted of college-aged American women [*N* = 25]) in Study 2 by Ketay et al. (2019), the present study more clearly demonstrated that the overlap between the self and others in the visual processing domain also occurs in a sample of college-aged East Asian women from Japan and China. The present study also revealed a condition-specific group difference in threshold. Japanese participants, compared with Chinese participants, needed a higher proportion of their own face to reach the chance-level (50%) response when their face was morphed with that of an unknown same-sex other, while no group difference was observed in the self–friend condition. This pattern suggests that Japanese participants may require a greater proportion of self-face information when their faces are morphed with those of unknown persons of the same sex. One possible interpretation is that the higher threshold shown by Japanese participants in the self–unknown condition may be related to previously reported cross-national differences in general trust, with average levels reported to be lower in Japan than in China (Ozono, 2019; Wike & Holzwart, 2008). This condition-specific group difference is consistent with our a priori expectation regarding cultural modulation in the self–unknown condition. Talhelm et al. (2014) reported that people in the rice-growing regions of China have several markers of East Asian culture (more holistic thoughts, more interdependent self-construals, and lower divorce rates), while those in the wheat-growing regions are more culturally similar to the West (more analytical thoughts, individualism, and higher rates of divorce). In the future, it will be necessary to pay attention to the participants’ place of origin when collecting data from samples of Chinese people, to facilitate more robust, detailed comparisons between Chinese and Japanese people.

An additional multiple linear regression analysis revealed that the threshold in the self–unknown morphing condition was significantly modulated by social skill (one of the subcategories of the AQ questionnaire), while the threshold in the self–friend morphing condition was not modulated. Specifically, the participants who had high levels of social skill (a low score in this subcategory) showed lower thresholds in the self–friend morphing condition than in the self–unknown morphing one (see each element in Fig. 3), suggesting that decreasing the ratio of one’s own image denotes an inclusion of others in the self in the self–friend morphing condition. The participants who had lower levels of social skill (a high score in this subcategory) showed comparable thresholds in both the self–friend and self–unknown morphing conditions. Based on the results of these comparable thresholds, it is difficult to determine whether the inclusion of others in the self occurs in participants with low social skill. Rather, the results might be related to the “egocentric bias” (i.e., difficulties in flexible switching between representations of the self and others) observed in autistic individuals (Lavenne-Collot et al., 2023).

The present results, showing comparable thresholds in participants with low social skill, possibly imply a performance invariance when these participants recognize their own and others’ faces. While Davis et al. (2017) used the Cambridge Face Memory Task (Duchaine & Nakayama, 2006) to demonstrate that female participants with lower social skill in the general population perform poorly on a face recognition task (Valla et al., 2013, but see also Rhodes et al., 2013), the present study revealed a novel finding by demonstrating that the modulation pattern of the threshold (regarded as the index of self-face representation, cf. Tsakiris, 2008) by social skill varies depending on the degree of familiarity of the morphing pairs of face photos in the self–other discrimination task.

As for the width, no significant correlations with the AQ subcategory were found in either the self–friend or self–unknown morphing conditions. The present results pertaining to width are consistent with and extend the studies by Chakraborty and Chakrabarti (2015, 2018), which analyzed the relationship between the slope (which is inversely proportional to the width) and the AQ score in a task similar to the self–unknown morphing condition of our task and found no significant correlation between slopes and AQ scores (they did not analyze the relationship between thresholds and AQ scores). In line with the current results, Uddin et al. (2008) also reported that the accuracy of each morph level between typically developing (*N* = 12, average age 12.23 ± 2.10) and ASD (*N* = 12, average age: 13.19 ± 2.61) participants had no significant difference, although they did not calculate the slope itself. Thus, the width that can be regarded as the boundary between the self and another seems not to be modulated by any subcategory of the AQ under either of our two morphing conditions.

While the present study provides novel insights into self–other discrimination among East Asian female university students using morphed face stimuli, several limitations should be recognized. Regarding the sample for this study, all participants were female, and all Chinese participants were students studying in Japan. In addition to recruiting male participants, future studies should recruit Chinese participants residing in China, taking into account the trait differences between regions there (i.e., rice- and wheat-growing regions), as pointed out by Talhelm et al. (2014). A further limitation is that individual differences were assessed only with the AQ. Future studies would benefit from including face-specific individual-difference measures, such as the 20-item Prosopagnosia Index (PI20; original: Shah et al., 2015; Japanese version: Nakashima et al., 2019; Traditional Chinese version: Wang et al., 2025), alongside the AQ, to clarify whether self–other discrimination in face recognition is more closely related to autistic traits, self-reported face-recognition ability, or both.

As described in the Materials and Methods section, all participants in this study first completed the self–friend condition, followed by the self–unknown condition, meaning the effect of presentation order could not be ruled out. As an exploratory supplementary analysis, we also examined accuracy at the two unambiguous endpoints of the morph continuum (0% self and 100% self), and found no effect of morphing condition at either endpoint (see Supplementary Information 1). While this result does not eliminate possible order-related effects, it suggests that the second (self–unknown) condition was not associated with an overall decline in performance. Since it has been demonstrated that self-face representation can be altered through experimental manipulation (Colonnello et al., 2013; Tsakiris, 2008), the possibility that self-face representation could be altered by presentation order and how such an alteration itself might affect task performance needs further investigation. Future studies should therefore counterbalance the order of conditions across participants or interleave the two conditions in a block design. Using eye-tracking techniques like those used in previous studies (e.g., Chakraborty & Chakrabarti, 2018; Davis et al., 2017; Fukui et al., 2021) will also be essential in the future, to examine in detail the properties of gaze behavior according to autistic traits in self–other discrimination during face recognition.

## Conclusion

This study investigated self–other discrimination in face recognition tasks completed by East Asian (i.e., Japanese and Han Chinese) female students using morphed self–friend and self–unknown images. We demonstrated that the inclusion of others in the self and the boundary between the self and others are modulated by personal familiarity, and that a condition-specific cultural difference also emerged, with Japanese participants showing a higher threshold than Chinese participants in the self–unknown condition. Furthermore, we found that the impact of personal familiarity on performance appears to vary depending on the extent of participants’ social skill (assessed via the AQ subscale).

## Supplementary Information

Below is the link to the electronic supplementary material.


Supplementary Material 1 (DOCX 66.9 KB)


## Data Availability

The data that support the findings of this study are available from the corresponding author upon reasonable request.
