# Supplementary material for: Self–other discrimination in face recognition depending on personal familiarity: investigating a sample consisting of Japanese and Han Chinese women
Source: Psychol Res. 2026 Jul 6;90(4):129. doi: 10.1007/s00426-026-02335-0 (PMC13337850; doi:10.1007/s00426-026-02335-0)
Supplement: Supplementary file 1 — Supplementary Material 1 (DOCX 66.9 KB) [file 426_2026_2335_MOESM1_ESM.docx]

Supplementary analysis using the evenly spaced 11-level subset (0–100% self in 10% increments)

To examine whether the main findings depended on the uneven spacing of the original 15 morph levels, threshold and width were re-estimated using an evenly spaced 11-level subset (0–100% self in 10% increments).

Threshold

We found a significant main effect of the morphed face stimuli [*F*(1, 33) = 40.436, *p* < 0.001, partial *η*^2^ = 0.551], with a lower threshold for the self–friend condition (*M* = 54.5, *SD* = 10.0) than for the self–unknown condition (*M* = 63.2, *SD* = 8.2), while no significant main effect of the group was noted [*F*(1, 33) = 1.529, *p* = 0.225, partial *η*^2^ = 0.044] between the Japanese (*M* = 60.5, *SD* = 9.4) and Chinese (*M* = 57.1, *SD* = 6.4) groups. An additional one-way ANCOVA including age as a covariate indicated that this non-significant main effect of the group remained unchanged after controlling for age [*F*(1, 32) = 2.137, *p* = 0.154]. The interaction between the group and the morphed face stimuli was significant [*F*(1, 33) = 5.341, *p* = 0.027, partial *η*^2^ = 0.139]. Post-hoc comparisons with the Bonferroni correction (α = 0.025) revealed a significantly larger threshold in the Japanese group (*M* = 66.4, *SD* = 8.9) than in the Chinese group (*M* = 59.9, *SD* = 5.9) for the self–unknown condition (*p* = 0.017), whereas no significant group difference was observed in the self–friend condition between the Japanese (*M* = 54.7, *SD* = 11.5) and Chinese (*M* = 54.4, *SD* = 8.6) groups (*p* = 0.941).

Width

We found a significant main effect of the morphed face stimuli [*F*(1, 33) = 16.030, *p* < 0.001, partial *η*^2^ = 0.327], with a larger width for the self–friend condition (*M* = 37.1, *SD* = 13.0) than for the self–unknown condition (*M* = 28.6, *SD* = 9.5), but a main effect of the group was not significant between the Japanese (*M* = 32.3, *SD* = 9.6) and Chinese (*M* = 33.4, *SD* = 9.7) groups [*F*(1, 33) = 0.128, *p* = 0.722, partial *η*^2^ = 0.004]. An additional one-way ANCOVA including age as a covariate indicated that this non-significant main effect of the group remained unchanged after controlling for age [*F*(1, 32) = 0.574, *p* = 0.454]. The interaction between the group and the morphed face stimuli was not significant [*F*(1, 33) = 0.166, *p* = 0.687, partial *η*^2^ = 0.005]. Mean width values were 36.9 (*SD* = 14.3) in the Japanese self–friend condition, 27.6 (*SD* = 8.3) in the Japanese self–unknown condition, 37.3 (*SD* = 12.0) in the Chinese self–friend condition, and 29.6 (*SD* = 10.8) in the Chinese self–unknown condition.

Pearson correlations between the 15-level and 11-level estimates

Pearson correlation coefficients were calculated between the estimates obtained from the original 15-level analysis and those obtained from the supplementary 11-level analysis.

Threshold estimates from the 15-level and 11-level analyses were highly correlated in both the self–friend (*r* = 0.991, *p* < 0.001) and self–unknown (*r* = 0.990, *p* < 0.001) conditions. Width estimates were also significantly correlated in both the self–friend (*r* = 0.830, *p* < 0.001) and self–unknown (*r* = 0.839, *p* < 0.001) conditions, although the correlations were lower than those observed for threshold (see Figure S2).

Exploratory supplementary analysis of accuracy at the unambiguous endpoints (0% self and 100% self)

To explore whether the fixed order of conditions might have been associated with a general decline in performance during the later session, we examined accuracy at the two unambiguous endpoints of the morph continuum (0% self and 100% self). Accuracy at each endpoint was analyzed using a two-way ANOVA with group (Japanese, Chinese) as a between-participants factor and morphing condition (self–friend, self–unknown) as a within-participants factor.

0% self

A two-way ANOVA revealed a significant main effect of group [*F*(1, 33) = 4.915, *p* = 0.034, partial *η*^2^ = 0.130], indicating lower accuracy in the Chinese group (*M* = 0.982, *SD* = 0.034) than in the Japanese group (*M* = 0.998, *SD* = 0.011). The main effect of the morphing condition was not significant [*F*(1, 33) = 0.641, *p* = 0.429, partial *η*^2^ = 0.019]. Mean accuracy was 0.989 (*SD* = 0.025) in the self–friend condition and 0.992 (*SD* = 0.027) in the self–unknown condition. The interaction between the group and morphing condition was also not significant [*F*(1, 33) = 0.001, *p* = 0.982, partial *η*^2^ < 0.001]. Mean accuracy values were 0.996 (*SD* = 0.016) in the Japanese self–friend condition, 1 (*SD* = 0) in the Japanese self–unknown condition, 0.980 (*SD* = 0.031) in the Chinese self–friend condition, and 0.984 (*SD* = 0.038) in the Chinese self–unknown condition.

100% self

Neither factor showed a significant main effect: group [*F*(1, 33) = 0.360, *p* = 0.553, partial *η*^2^ = 0.011], morphing condition [*F*(1, 33) = 2.588, *p* = 0.117, partial *η*^2^ = 0.073]. Mean accuracy was 0.985 (*SD* = 0.032) in the Japanese group and 0.980 (*SD* = 0.031) in the Chinese group, and 0.989 (*SD* = 0.030) in the self–friend condition and 0.977 (*SD* = 0.032) in the self–unknown condition. The interaction between the group and morphing condition was also not significant [*F*(1, 33) = 0.245, *p* = 0.624, partial *η*^2^ = 0.007]. Mean accuracy values were 0.993 (*SD* = 0.031) in the Japanese self–friend condition, 0.978 (*SD* = 0.032) in the Japanese self–unknown condition, 0.984 (*SD* = 0.029) in the Chinese self–friend condition, and 0.977 (*SD* = 0.033) in the Chinese self–unknown condition.


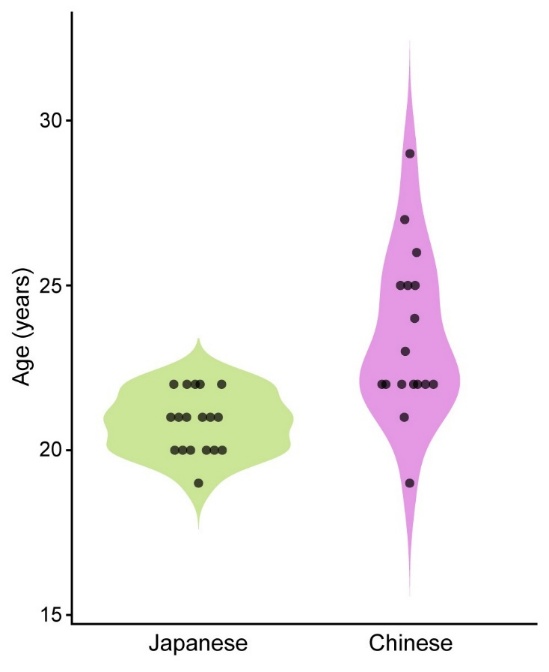


Figure S1. Age distributions of the Japanese and Chinese groups.

Figure S2. Correlations between estimates obtained from the original 15-level analysis and the supplementary 11-level analysis.

Table S1 Deviance per block and the overdispersion parameter *η* for all participants in the self–friend and self–unknown conditions.

|  |  | deviance per block | |  | *η* |  |
| --- | --- | --- | --- | --- | --- | --- |
|  | ID | Self–friend | Self–unknown |  | Self–friend | Self–unknown |
| Japanese | 1 | 0.50 | 0.53 |  | 4.40E-13 | 1.15E-15 |
|  | 2 | 0.64 | 0.54 |  | 2.43E-16 | 1.24E-15 |
|  | 3 | 0.60 | 0.12 |  | 9.33E-16 | 8.78E-16 |
|  | 4 | 1.30 | 0.85 |  | 1.39E-15 | 1.40E-15 |
|  | 5 | 0.93 | 0.29 |  | 2.84E-15 | 0.00E+00 |
|  | 6 | 0.07 | 0.02 |  | 8.90E-16 | 1.48E-04 |
|  | 7 | 0.71 | 0.63 |  | 2.60E-09 | 1.08E-14 |
|  | 8 | 0.70 | 0.63 |  | 1.11E-14 | 3.47E-16 |
|  | 9 | 1.52 | 0.48 |  | 5.87E-15 | 1.64E-15 |
|  | 10 | 0.38 | 0.92 |  | 3.41E-12 | 1.93E-15 |
|  | 11 | 1.41 | 0.17 |  | 1.77E-15 | 9.59E-16 |
|  | 12 | 0.68 | 0.48 |  | 1.80E-04 | 3.56E-05 |
|  | 13 | 1.65 | 0.40 |  | 1.62E-15 | 9.35E-07 |
|  | 14 | 0.20 | 0.48 |  | 1.13E-07 | 1.79E-15 |
|  | 15 | 1.19 | 0.12 |  | 1.78E-15 | 1.16E-04 |
|  | 16 | 0.51 | 0.79 |  | 2.13E-15 | 2.86E-15 |
|  | 17 | 0.59 | 0.74 |  | 1.10E-04 | 1.90E-16 |
|  | 18 | 0.44 | 0.37 |  | 6.23E-16 | 9.16E-16 |
| Chinese | 1 | 1.23 | 0.44 |  | 1.20E-15 | 2.82E-06 |
|  | 2 | 1.20 | 0.89 |  | 1.01E-15 | 1.51E-15 |
|  | 3 | 0.97 | 1.06 |  | 5.44E-14 | 4.17E-15 |
|  | 4 | 0.89 | 0.64 |  | 1.26E-15 | 2.60E-15 |
|  | 5 | 0.24 | 0.62 |  | 1.94E-08 | 1.83E-16 |
|  | 6 | 0.89 | 0.91 |  | 1.01E-14 | 9.90E-16 |
|  | 7 | 0.68 | 0.44 |  | 2.09E-16 | 2.98E-12 |
|  | 8 | 0.44 | 0.12 |  | 2.92E-13 | 1.16E-04 |
|  | 9 | 0.66 | 0.41 |  | 1.01E-16 | 1.25E-11 |
|  | 10 | 0.75 | 0.31 |  | 1.33E-15 | 2.31E-16 |
|  | 11 | 0.79 | 0.70 |  | 2.53E-13 | 4.92E-16 |
|  | 12 | 0.42 | 0.52 |  | 3.98E-16 | 1.26E-15 |
|  | 13 | 0.63 | 0.82 |  | 1.26E-08 | 1.27E-15 |
|  | 14 | 0.50 | 0.80 |  | 7.96E-08 | 3.08E-16 |
|  | 15 | 0.48 | 0.93 |  | 1.64E-15 | 1.83E-15 |
|  | 16 | 0.59 | 1.00 |  | 7.46E-05 | 1.35E-11 |
|  | 17 | 0.68 | 1.34 |  | 4.36E-16 | 2.88E-15 |
|  |  |  |  |  |  |  |
| Max. |  | 1.65 | 1.34 |  | 1.80E-04 | 1.48E-04 |
| Min. |  | 0.07 | 0.02 |  | 1.01E-16 | 0.00E+00 |
| Median |  | 0.68 | 0.54 |  | 2.13E-15 | 1.64E-15 |
